# Interactions of Boron Clusters and their Derivatives with Serum Albumin

**DOI:** 10.1038/s41598-017-10314-0

**Published:** 2017-08-29

**Authors:** Tomasz M. Goszczyński, Krzysztof Fink, Konrad Kowalski, Zbigniew J. Leśnikowski, Janusz Boratyński

**Affiliations:** 1 Laboratory of Biomedical Chemistry, Department of Experimental Oncology, Hirszfeld Institute of Immunology and Experimental Therapy, Polish Academy of Sciences, 12 Rudolf Weigl St., 53-114 Wrocław, Poland; 2 Laboratory of Molecular Virology and Biological Chemistry, Institute of Medical Biology, Polish Academy of Sciences, 106 Lodowa St., 93-232 Łódź, Poland

## Abstract

Boron clusters are polyhedral boron hydrides with unique properties, and they are becoming increasingly widely used in biology and medicine, including for boron neutron capture therapy (BNCT) of cancers and in the design of novel bioactive molecules and potential drugs. Among boron cluster types, icosahedral boranes, carboranes, and metallacarboranes are particularly interesting, and there is a need for basic studies on their interaction with biologically important molecules, such as proteins. Herein, we report studies on the interaction of selected boron clusters and their derivatives with serum albumin, the most abundant protein in mammalian blood. The interaction of boron clusters with albumin was examined by fluorescence quenching, circular dichroism, dynamic and static light scattering measurements and MALDI-TOF mass spectrometry. Our results showed that metallacarboranes have the strongest interaction with albumin among the tested clusters. The observed strength of boron cluster interactions with albumin decreases in order: metallacarboranes [M(C_2_B_9_H_11_)_2_]^−^ > carboranes (C_2_B_10_H_12_) >> dodecaborate anion [B_12_H_12_]^2−^. Metallacarboranes first specifically interact with the binding cavity of albumin and then, with increasing compound concentrations, interact non-specifically with the protein surface. These findings can be of importance and are useful in the development of new bioactive compounds that contain boron clusters.

## Introduction

There is a growing interest among researchers and in the pharmaceutical industry in the use of boron as a component of bioactive molecules. An important class of boron compounds is polyhedral boron hydrides (boron clusters), which have a non-planar, cage-like structure. Boron clusters are man-made molecules with remarkable properties, such as chemical, biological and thermal stability; low toxicity; high (depending on the structure) hydrophilicity, hydrophobicity or amphiphilicity; three-dimensional delocalization of cluster electrons; high skeleton rigidity; and the ability to form dihydrogen σ-hole bonding^1–3^. Boron clusters have been introduced to molecules with diverse biological activity, where they serve as pharmacophores or modulators of the physicochemical and biological properties of the mother compounds^4^. Furthermore, they can be used as physiologically inert boron carriers in boron neutron capture therapy (BNCT) and diagnostics^5–7^. The boron clusters that are the most commonly used in medicinal chemistry are as follows: negatively charged 3-cobalt-bis(1,2-dicarbollide)ate [Co(C_2_B_9_H_11_)_2_^−^], neutral dicarba-*closo*-dodecaboranes (C_2_B_10_H_12_), and double charged dodecaborate ([B_12_H_12_]^2−^)^4^.

Metallacarborane clusters are built from two dicarbollide subclusters that sandwich a central metal atom. The best known example of this group of compounds, cobalt bisdicarbollide, [3,3′-Co(1,2-C_2_B_9_H_11_)_2_]^−^, [CoD]^−^, is a boron-based anion consisting of a central cobalt atom placed between two carboranyl clusters^8^. [CoD]^−^, as a result of its dispersed net negative charge and hydrophobic surface, possesses amphiphilic characteristics and is soluble in both water and organic solvents. In addition, its weakly polarized B-H and C-H bonds^9^ are responsible for the intermolecular interactions of [CoD]^−^, which lead to aggregation and micelle formation in aqueous solutions^10–12^. It has been reported that [CoD]^−^ behaves similarly to anionic surfactants in aqueous solutions^13^ and can form well organized, fairly monodisperse structures^10^. Studies performed by Rak *et al*., designed to identify agents that would stabilize [CoD]^−^ in water, found that human serum albumin (HSA) is one of the best excipients^14^. NMR studies showed the formation of complexes with high stoichiometry caused by nonspecific binding of the metallacarborane on surface of HSA^15^. The interaction between [CoD]^−^ and HSA is mainly driven by hydrophobic forces because the strength of this interaction is correlated with the lipophilicity (log P_OW_) of the metallacarborane derivatives^16^. [CoD]^−^ also binds to the hydrophobic pocket of HIV protease and acts as a potent, specific and selective inhibitor of that enzyme^17, 18^.

The dicarba-*closo*-dodecaboranes (carboranes) are hydrophobic, icosahedral carbon-containing boron clusters with an approximate volume occupied by carbaboranes about 40% larger than that of rotating benzene ring. They exist in the following three isomeric forms: *closo*-1,2-C_2_B_10_H_12_ (*ortho*-carborane), *closo*-1,7-C_2_B_10_H_12_ (*meta*-carborane) and *closo*-1,12-C_2_B_10_H_12_ (*para*-carborane). Carboranes introduced into the structures of bioactive compounds usually replace hydrophobic components, such as a phenyl ring or adamantane, and enhance hydrophobic interactions with the receptors of those compounds^19, 20^. Furthermore, biomolecules modified with boron clusters are often more resistant to degradation, increasing the stability and bioavailability of bioactive molecules^21^. Due to these advantages, carboranes have been used as pharmacophores in nonsteroidal anti-inflammatory drugs^22^; anti-folates^23^; carbonic anhydrase inhibitors^24^; thrombin inhibitors^25^; hypoxia-inducible factor (HIF) inhibitors^20, 26^; purinergic receptor ligands^27–29^; analogues of a local anesthetic, lidocaine^30^; antiviral drugs^31, 32^ and other bioactive molecules.

Dodecaborate is an icosahedral, dianionic, highly water soluble boron cluster. Although hydrophilic, dodecaborate forms strong inclusion complexes with γ-cyclodextrin^33^. The interaction with the hydrophobic cavity of cyclodextrin is driven by a chaotropic effect and not by hydrophobic forces. Derivatives of dodecaborate interact with lipid membranes^34^. Due to its high boron content, dodecaborate is commonly used as a boron carrier for BNCT.^10^B-sodium-mercaptoundecahydrododecaborate (BSH)^35^ and ^10^B-L-borophenylalanine^36^ are used as boron carriers in BNCT^35^.

Almost every drug, theranostic or diagnostic agent interacts with albumin, the most abundant protein in mammalian blood. Serum albumin (SA) possesses binding ability for a broad spectrum of compounds, either endogenous and exogenous, due to the numerous binding regions and diverse specificity^37^. Bovine serum albumin (BSA) has three primary binding sites for long-chain fatty acids (one site in domain I and two sites in domain III) and two secondary sites^38–40^. With respect to exogenous compounds, BSA has two high affinity binding sites for drugs, known as the Sudlow’s sites I and II, which are located in domains II and III^41^. Binding with serum proteins influences the pharmacokinetics and pharmacodynamics of these compounds^42, 43^. Hence, investigation of the binding affinity of boron clusters to SA may provide useful information for designing novel boron-containing, biologically active molecules.

To the best of our knowledge, except for the use of HSA as a solubilizing agent for metallacarboranes^14, 15^, there are no studies describing the interactions of boron clusters (and molecules containing these compounds) with serum proteins. The aim of this research is to address this gap and to characterize interactions between bovine serum albumin and various boron clusters. Boron clusters of three different types were selected for the study due to differences in their physicochemical properties and applications in medicinal chemistry.

Herein, the interactions of boron clusters and hybrid organic-boron cluster conjugates with BSA were investigated with fluorescence, absorption and circular dichroism spectroscopy as well as dynamic and static light scattering and mass spectrometry techniques. The pattern of the interactions with BSA and an effect of the boron clusters type on binding to the BSA molecule were established.

## Results and Discussion

### Fluorescence quenching mechanism

BSA, because of its structural homology to human serum albumin (HSA), is often used to study interactions with drugs^37^. No major differences between HSA and BSA have been observed in terms of the binding constant or binding mode^44, 45^. The intrinsic fluorescence of BSA comes from tryptophan (Trp), tyrosine (Tyr) and phenylalanine (Phe) residues. However, the Trp residue has the strongest fluorescence intensity and is the most sensitive to changes in the microenvironment; therefore, it is indicative of the protein conformational alterations with binding. BSA has two Trp residues (Trp134 and Trp212), which are located within hydrophobic pockets in the first and second domains of BSA molecule^46^. Upon binding of a ligand in these pockets, the Trp environment changes, quenching the fluorescence.

**Figure 1:**
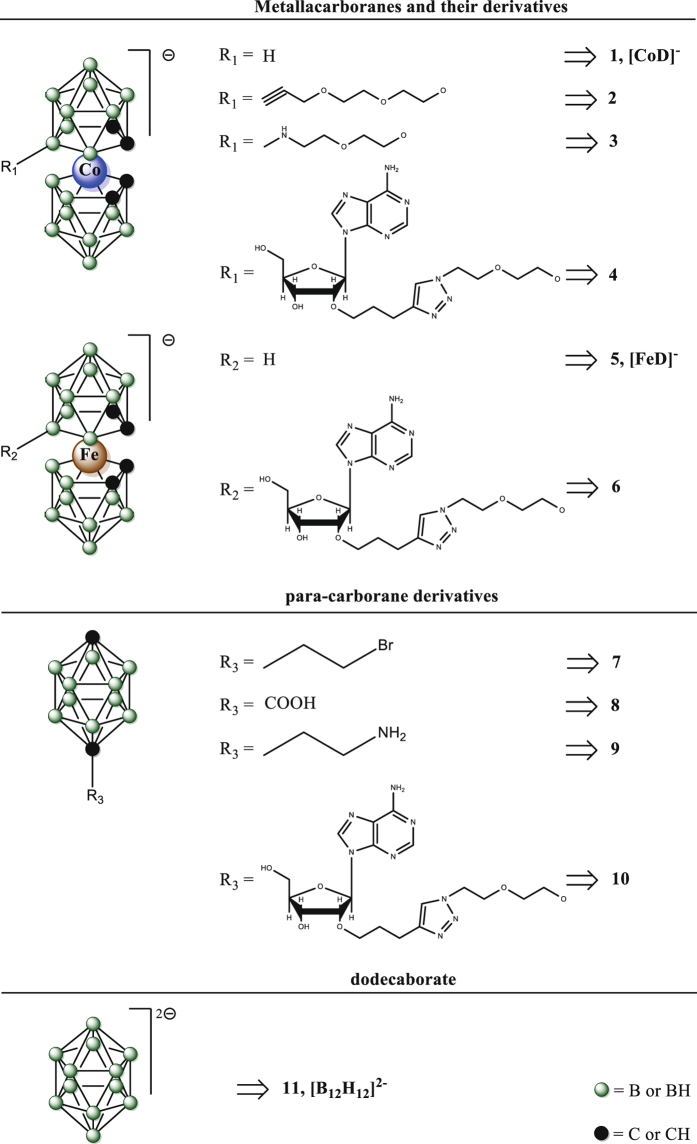
Structures of boron clusters and their derivatives.

**Figure 2:**
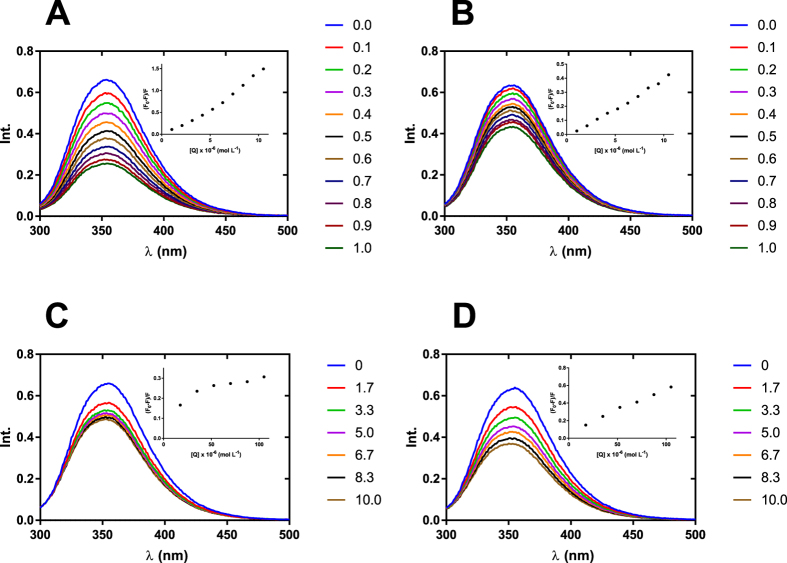
Fluorescence emission spectra of BSA (10.5 μM) in the presence of: [CoD]^−^ – **1** (**A**), **4** (**B**), **7** (**C**) and **10** (**D**) in 0.10 M sodium bicarbonate (pH 8.4) with 2% DMSO, *λ*_*ex*_ = 280 nm, 298 K. The concentration of **1** and **4** was 0 to 10.5 μM with 1.05 μM intervals; the concentration of **7** and **10** was 0 to 105 μM with 17.5 μM intervals. Detailed molar excess of boron cluster is described in the legend. Inset: Stern-Volmer plots for BSA – boron cluster complexes.

In the first step, we measured the fluorescence intensity of BSA in the presence of boron clusters of three different types, metallacarborane (**1**); *para*-carborane derivative (**7**) and dodecaborate (**11**) (Fig. 1). The BSA: boron clusters molar ratio was 1 : 1. The parameter degree of quenching was calculated (Table S1). Based on these results, we decided to perform further measurements of the fluorescence intensity of BSA in the presence of metallacarboranes (compounds **1** to **6**) with a maximum BSA : boron cluster molar ratio of 1 : 1 and to increase the ratio of the other boron clusters (**7** to **11**) to 1 : 10 (Fig. 2, Fig. S1).

The fluorescence quenching mechanism can be divided into the following two mechanisms: dynamic quenching, caused by collisional encounters of fluorophores and quenchers, or static quenching, caused by ground-state complex formation between fluorophores and quenchers^47^. Dynamic and static quenching can be distinguished by their dependence on temperature and excited-state lifetime. If the value of the quenching constant (*K*_*SV*_) decreases with the increasing temperature, the mechanism is usually static because the higher temperature is likely to decrease the stability of the complex. If *K*_*SV*_ increases with increasing temperature, the mechanism is likely dynamic because the higher temperature results in a larger diffusion coefficient and promotes electron transfer. The quenching constant (*K*_*SV*_) was calculated using the Stern-Volmer equation (1)^47^:

1 $$\frac{{F}_{0}-F}{F}={K}_{SV}[Q]={k}_{q}{\tau }_{0}[Q]$$

where *F*_0_ and *F* are the steady-state fluorescence intensities in the absence and presence of quencher, respectively, and [*Q*] denotes the initial concentration of quencher. *k*_*q*_ is the quenching rate constant of BSA and *τ*_0_ is the fluorescence lifetime of BSA in the excited state without any quencher, and its value is 6×10^−9^ s^48^. *K*_*SV*_ can be calculated from the slope of the Stern-Volmer equation (Fig. S2).

To determine the mechanism of fluorescence quenching in the case of boron clusters and BSA, the measurements of fluorescence quenching were performed at three temperatures (25, 31 and 37 °C). We observed differences among different types of boron clusters. Hydrophobic metallacarboranes (**1** to **6**) were characterized by the highest value of *K*_*SV*_ on the order of 10^4^ to 10^5^ (Table S2). For doubly negatively charged dodecaborate **11**, the fluorescence quenching was not observed. For neutral *para*-carborane derivatives (**7** to **9**), nonlinear dependence of quencher concentration and fluorescence quenching were observed, except for the conjugate of carborane and adenosine, where the correlation was linear. Therefore, the *K*_*SV*_ value could not be calculated for the last two boron cluster types. For metallacarboranes (**1** to **6**), we observed an increase of the *K*_*SV*_ value with increasing temperature, which indicates a dynamic mechanism of quenching. However, the values of the quenching rate constants (*k*_*q*_) are 2- to 3-fold higher than the maximum scatter collision quenching constant for various quenchers with biopolymers (10^10^ L mol^−1^ s^−1^)^49^, suggesting that static quenching also occurs.

### Binding constant and binding modes

For the boron cluster – BSA interaction, the binding constant (*K*_*b*_) and number of binding sites (n) of the complexes can be calculated by the equation (2), under assumption that BSA has independent binding sites^47^.

2 $$\mathrm{log}\,\frac{{F}_{0}-F}{F}=\,\mathrm{log}\,{K}_{b}+n\,\mathrm{log}\,[Q]$$

The *K*_*b*_ and *n* values can be calculated by the intercept and slope of the regression curve (Fig. S3). The estimated *K*_*b*_ values were on the order of 10^5^ M^−1^ (Table S3) for metallacarboranes (**1** to **6**), which is similar to the values of the binding constant for small organic drugs that have a strong binding force with BSA, such as aspirin^41^. The values of *n* were approximately equal to 1 in the studied temperature range, indicating the existence of a single binding site of boron clusters on BSA.

In the binding process for drugs with proteins, several binding forces can be engaged, including hydrogen bonding interaction, van der Waals forces, electrostatic interaction and hydrophobic interaction. The distinction between those forces can be made based on the signs and magnitudes of the thermodynamic parameters, such as the Gibbs free energy change (*ΔG* ^0^), enthalpy change (*ΔH* ^0^) and entropy change (*ΔS* ^0^). The thermodynamic parameters can be calculated by the equations (3) and (4).

3 $${\rm{In}}\,{{K}}_{b}=-\frac{\Delta {H}^{0}}{RT}+\frac{\Delta {S}^{0}}{R}$$

4 $$\Delta {G}^{0}=-RT\,{\rm{In}}\,{K}_{b}$$

where *R* is the gas constant and *K*_*b*_ is the binding constant at corresponding temperatures (Fig. S4). Based on the thermodynamic view point^50^, the following three possible scenarios can be envisioned: (I) positive values of both *ΔH* ^0^ and *ΔS* ^0^ for the hydrophobic interaction, (II) negative values of both *ΔH* ^0^ and *ΔS* ^0^ for the van der Waals and/or hydrogen bonding interaction, and (III) *ΔH* ^0^ close to 0 and positive value of *ΔS* ^0^ for the electrostatic interaction.

The positive values of both *ΔH* ^0^ and *ΔS* ^0^ for **1** to **6** (Table S3) suggest that the interaction mode for metallacarboranes binding on BSA is mainly a hydrophobic interaction. This result is in agreement with studies by Rak *et al*. in which the strength of the interaction with BSA was correlated with the lipophilicity of metallacarborane derivatives, suggesting the hydrophobic nature of the interaction^16^. Moreover, the negative value of *ΔG* ^0^ suggests that binding of the boron clusters with BSA is a spontaneous process.

Rak *et al*. reported a non-specific interaction of (**1**) derivatives with HSA for complexes with high stoichiometry^15^. Our studies supplement those observations with studies of complexes that have low stoichiometry with a maximum ratio of metallacarborane : BSA set to 1 : 1. In contrast to the previous report, we have found that under low stoichiometry conditions, metallacarboranes have a specific interaction with BSA by binding in the hydrophobic cavity of the protein. These observations lead to hypothesis that at low stoichiometry, metallacarboranes interact specifically with the hydrophobic cavity of albumin, while at high stoichiometry, they also interact non-specifically with the protein surface.

For further studies, we have chosen [CoD]^−^ (**1**) and [B_12_H_12_]^2−^ (**11**) as compounds with the strongest and weakest interactions with BSA, respectively. In that way, we could study the influence of the boron cluster types on the interaction with BSA in two extreme cases. Incorporation of [CoD]^−^ into the structure of bioactive molecule can significantly affect its interactions with serum albumin. By contrast, [B_12_H_12_]^2−^ does not seem to affect the affinity of the conjugate towards albumin. Furthermore, in contrast to the other two types of boron clusters, the number of studies on the interaction of [B_12_H_12_]^2−^ with proteins is very limited. Considering that derivatives of this boron cluster are used as boron carrier in BNCT, there is a gap in our knowledge that needs to be addressed.

### Conformational changes of BSA induced by boron clusters

**Figure 3:**
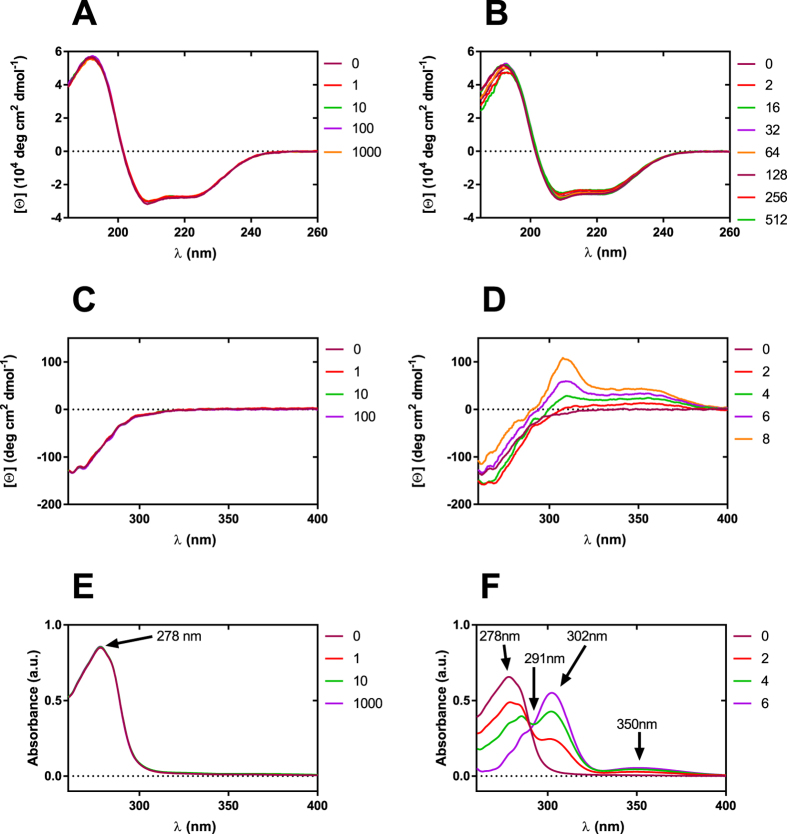
Circular dichroism and absorption spectra of BSA in the presence of increasing concentration of selected boron clusters. Far UV CD spectra, C(BSA) = 1.5 μM, C([B_12_H_12_]^2−^) in the range 0–1.5 mM (**A**), C(BSA) = 1.5 μM, C([CoD]^−^) in the range 0–1.5 mM (**B**); near UV CD spectra, C(BSA) = 150 μM, C([B_12_H_12_]^2−^) in the range 0–15 mM (**C**), C(BSA) = 150 μM, C([CoD]^−^) in the range 0–1.2 mM (**D**); UV absorption spectra, C(BSA) = 12.6 μM, C([B_12_H_12_]^2−^) in the range 0–12.6 mM (**E**), C(BSA) = 9.79 μM, C([CoD]^−^) in the range 0–58.7 μM (**F**). Detailed molar excess of boron cluster is described in the legend. Far UV CD spectra were measured in NaHCO_3_ 0.01 M and near UV CD and absorption spectra were measured in NaHCO_3_ 0.1 M.

The CD spectrum of native BSA (25 °C, NaHCO_3_ 10 mM) exhibited two negative minima at 208 and 222 nm and a maximum at 192 nm, which is in good agreement with previous studies^51^ and characteristic for proteins with high helical content. The CD spectra of native BSA in the presence of an increasing concentration of selected boron cluster (from 0 to 1000 molar excess) are represented in Fig. 3. The influence of [B_12_H_12_]^2−^ on the BSA structure revealed in the far UV CD spectra is negligible. No essential conformational changes were observed for BSA in the presence of [B_12_H_12_]^2−^ (0 to 1000 molar excess), indicating that the presence of the boron cluster cage did not significantly alter the tertiary structure of BSA (Fig. 3A).

The obtained data show that the portion of α-helices in the secondary structure of BSA is equal to 59% and increasing the concentration of [CoD]^−^ in the BSA environment leads to a slight decrease in the number of α-helices (up to 54% for 512 molar excess of the boron cluster – Fig. 3B). The observed changes in the content of α-helices caused by [CoD]^−^ are noticeable, but they seem to be negligible compared with changes in the BSA α-helix content caused by ionic surfactants such as SDS^51, 52^. This observation is of interest because [CoD]^−^ may behave like a classical ionic surfactant in selected cases, and it is able to form complexes with polyelectrolytes^12^. Features of near-UV CD spectra are more interesting. First, the presence of [B_12_H_12_]^2−^ (0 to 100 molar excess) does not disturb the CD spectrum (Fig. 3C). In the case of [CoD]^−^, the situation is radically different. The presence of a metallacarborane cluster (0–8 molar excess) leads to formation of two new CD signals (Fig. 3D). Two positive Cotton effects at 350 nm and 309 nm are directly connected with complex formation between [CoD]^−^ and the hydrophobic pocket of BSA. Slight changes in the CD spectrum below 300 nm suggest that this process does not significantly alter the tertiary structure of BSA. In addition, the formation of the [CoD]^−^ : BSA complex is shown in the absorption spectrum (Fig. 3F) where increasing the concentration of [CoD]^−^ causes the formation of two new absorption spectra (with a maximum at 350 nm and 302 nm) as well as the simultaneous decrease in the characteristic band for BSA at 278 nm and an isosbestic point at 291 nm.

### Hydrodynamic changes of BSA induced by boron clusters

The weak protein–protein interactions, i.e., non-specific interactions, were studied using a combination of DLS and SLS techniques. Here we used two parameters to describe the BSA-BSA interactions in the presence of selected boron clusters, i.e., diffusion interaction parameter (*k*_*D*_) and second virial coefficient (*B*_22_), both of which are first-order coefficients of protein interactions. The diffusion interaction parameter (*k*_*D*_) is a useful factor to study weak protein interactions and is connected to specific biophysical properties, such as the aggregation propensity. The diffusion coefficient (*D*) was measured as a function of the BSA concentration in the presence of a defined boron cluster using the DLS protocol. The value of *k*_*D*_ (mL g^−1^) was determined from the slope of a plot of *D* vs. the BSA concentration, where *D* ^0^ is the extrapolated intercept (single particle diffusivity) and the slope is *D* ^0^ *kD* (equation 5).

5 $$D={D}^{0}(1+{k}_{D}{C}_{BSA})$$

The second virial coefficient (*B*_22_), is another parameter for studying weak protein interactions. Positive *B*_22_ is interpreted as indicative of the presence of weak, net-repulsive forces between protein molecules, while negative *B*_22_ is considered to reflect net-attractive forces. The normalized Rayleigh ratio *R*_*θ*_ from the SLS measurement is related to the properties of the protein solution (equation 6).

6 $$\frac{KC}{{R}_{\theta }}={M}^{-1}+2{B}_{22}{C}_{BSA}$$

The value of *B*_22_ was determined from the slope of a plot of *KC/R* vs. the BSA concentration.

**Figure 4:**
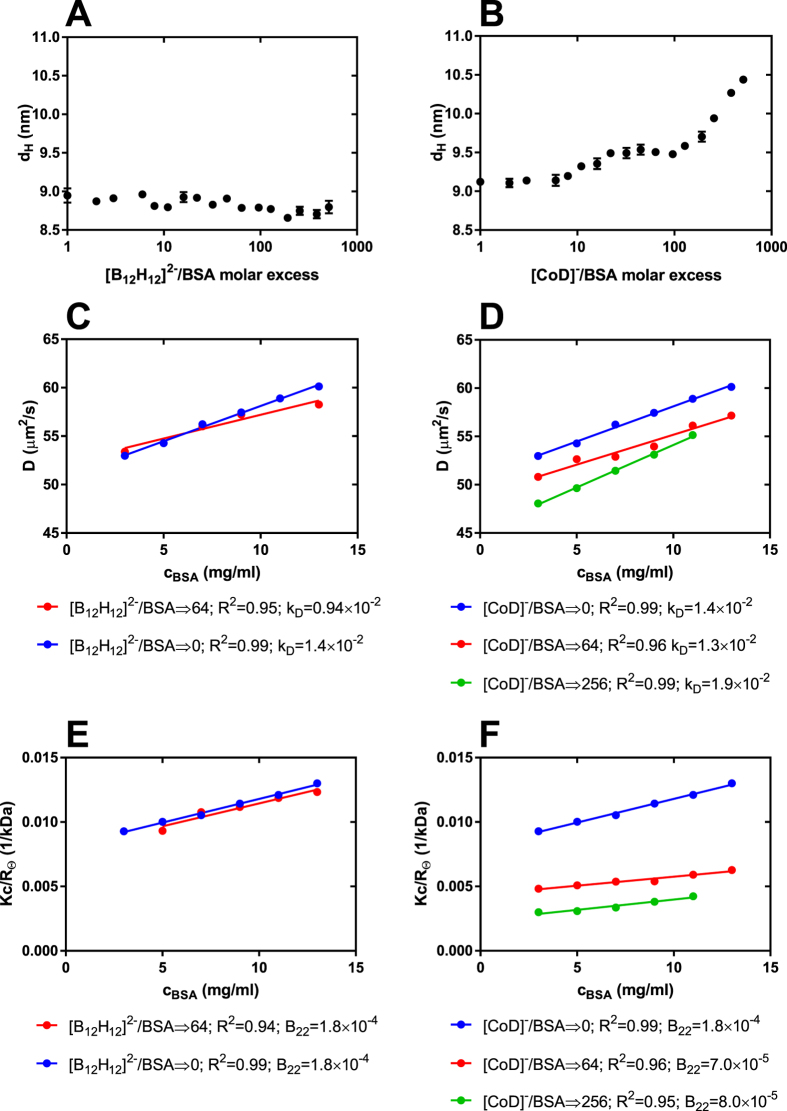
Boron cluster-specific effects on hydrodynamic parameters of BSA determined by the DLS method. Hydrodynamic diameter of BSA as a function of concentration of [B_12_H_12_]^2−^ (**A**) and [CoD]^−^ (**B**), there was constant concentration of BSA (15 μM) and boron clusters concentration were in the range 0–7.7 mM. Plot of diffusion coefficient of BSA as function of protein concentration, in the presence of [B_12_H_12_]^2−^ (**C**) and [CoD]^−^ (**D**), the value of diffusion interaction parameter (*k*_*D*_, g mL^−1^) was determined from the slope of the plot. Plot of the Debye ratios *KC/R*_*θ*_ of BSA as function of protein concentration, in the presence of [B_12_H_12_]^2−^ (**E**) and [CoD]^−^ (**F**), the value of second virial coefficient (*B*_22_, mL mol g^−2^) was determined from the slope of the plot.

Combining SLS and DLS techniques, we determined the boron cluster-specific effects on BSA hydration and on the hydrodynamic interactions among the BSA molecules. The DLS measurements at finite protein concentrations (15.1–197 μM) using both cluster types, [CoD]^−^ and [B_12_H_12_]^2−^, showed that the diffusion coefficient was affected by both direct and hydrodynamic interactions^53^. The presence of positive slopes in both DLS and SLS data in the case of two distinct boron clusters, together with the linear behavior of both data, support that the potential contributions of these boron clusters to protein aggregation are negligible (Fig. 4). In addition, in the case of [CoD]^−^: BSA, the intercepts in the DLS and SLS data indicate the hydrodynamic size and total molecular weight of the protein increases with increasing [CoD]^−^ concentration (unlike for [B_12_H_12_]^2−^), suggesting the formation of [CoD]^−^: BSA complexes with increasing numbers of attached metallacarborane clusters. The hydrodynamic diameter of BSA was independent of the [B_12_H_12_]^2−^ concentration (Fig. 4A), but [CoD]^−^ exerted a pronounced influence on the hydrodynamic size of BSA, as observed in Fig. 4B for a [CoD]^−^: BSA molar ratio higher than 10 for which the hydrodynamic diameter of BSA (or [CoD]^−^ : BSA complex) slightly increased up to 9.5 nm. Next, despite the increase in the concentration of metallacarborane, we observed a fixed hydrodynamic size. Subsequently, for molar ratios higher than 100, the hydrodynamic size of BSA again starts to increase, suggesting the appearance of second generation complexes. Considering the shape and dimensions of [CoD]^−^ (0.6×1.1 nm peanut shape), we can interpret these data as indicative of a non-specific interaction of [CoD]^−^ with the BSA surface, which gives rise to a first interaction layer followed by an increasing [CoD]^−^: BSA molar ratio over 100; then, metallacarborane clusters begin to form the second interaction layer.

Additional information on the behavior of BSA in the presence of boron clusters was obtained by DLS in the heat-treating experiment (Fig. S5). This method allows for registration of changes in the hydrodynamic size of the protein to detect aggregation. According to the literature reports^54, 55^, the hydrodynamic size of BSA should significantly increase for temperatures higher than 60 °C. For BSA in the absence of the boron clusters as well as for BSA in the presence of [B_12_H_12_]^2−^, our results are in good agreement with these reports. Sodium dodecaborate did not influence the hydrodynamic behavior of BSA during thermal treatment (Fig. S5C). A different situation was observed in the case of [CoD]^−^. Metallacarborane exerted a profound influence on BSA during heating when the temperature ranged from 25 °C to 85 °C, and this process depends on the [CoD]^−^ concentration (Fig. S5D). [CoD]^−^ can limit thermal-induced BSA aggregation and the resulting aggregates are much smaller than native BSA, which is also noticeable after cooling to room temperature. In addition, [CoD]^−^ influences the aggregation kinetics at a constant temperature of 60 °C (Fig. S5F). Selected cases have inhibition of aggregation at that temperature.

### MALDI-MS

**Figure 5:**
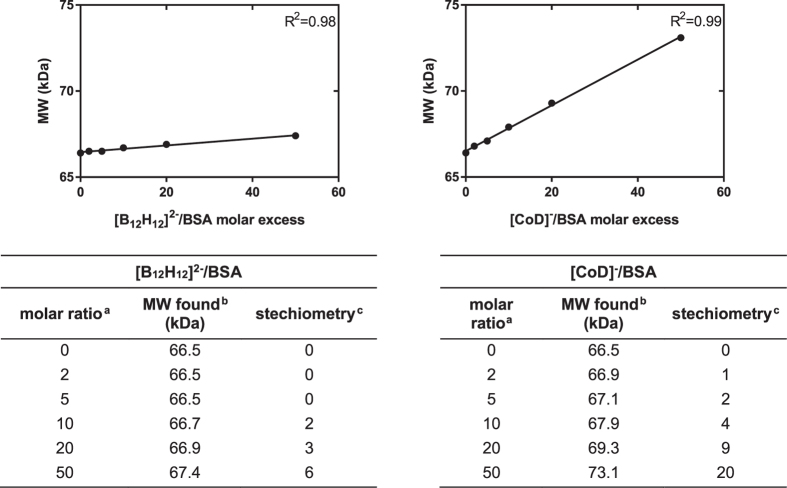
Mass spectrometry results of nonconvalent binding complexes of BSA and [B_12_H_12_]^2−^ (**A**) and [CoD]^−^ (**B**). ^a^Molar ratio of BSA (15 μM) and boron cluster placed on the MALDI plate, ^b^Molecular weight found in maldi, ^c^calculated complex stechiometry.

We also demonstrated the interactions of boron clusters with BSA using the MALDI-MS technique. A molecular mass measurement was made as a function of the boron cluster concentration and the stoichiometry of the protein : boron cluster complex was calculated from these data. The results of the formation of non-covalent binding complexes between [CoD]^−^ or [B_12_H_12_]^2−^ and BSA are summarized in Fig. 5. Both types of boron clusters can form non-covalent complexes with BSA. Moreover, the measured molecular weight increased linearly with the boron cluster concentration, allowing for the calculation of the stoichiometry of complexes formed. It is noteworthy that the measurement was made in acidic MALDI matrix, in the presence of organic solvent and that the conditions did not cause dissociation of the complexes. Changes in the molecular weight are even significant at a [CoD]^−^: BSA molar ratio of 2 : 1. This observation suggests that complex formation between [CoD]^−^ and BSA is very efficient and the resulting complexes very stable. In contrast, the interaction of [B_12_H_12_]^2−^ with BSA is much weaker (cf. experimental) and only visible at higher molar ratios.

## Conclusions

The interaction between BSA and three types of boron clusters (metallacarboranes, *para*-carboranes and dodecaborates) and their derivatives was studied using measurements of fluorescence quenching of BSA in increasing concentrations of the boron clusters and their derivatives. The observed strength of boron cluster interactions with BSA decreases in order: metallacarboranes [M(C_2_B_9_H_11_)_2_]^−^ > carboranes (C_2_B_10_H_12_) >> dodecaborate anion [B_12_H_12_]^2−^. Metallacarboranes and their derivatives (**1** to **6**) strongly interact with BSA. The strongest binding constant was obtained for [CoD]^−^. Covalent attachment of organic component to metallacarboranes does not significantly reduce the strength of the interaction with BSA. The conjugates of [CoD]^−^ and [FeD]^−^ with adenosine (**4** and **6**, respectively) interact with the hydrophobic cavity of albumin and have a similar strength as for metallacarboranes alone (**1** and **5**), which suggests that metallacarboranes can serve as anchors to albumin for drugs, prolonging their half-life. In addition, binding constants (K_b_) for metallacarborane with cobalt atom ([CoD]^−^, **1**) and metallacarborane with iron atom ([FeD]^−^, **5**) are similar, which suggests that coordinated metal atom does not significantly affect the affinity of metallacarboranes towards albumin.

Carboranes (**7** to **10**) also interact with BSA; however, the relationship between the boron cluster concentration and fluorescence quenching of BSA does not follow a linear correlation, except for the conjugates of carborane and adenosine (**10**). The results for [B_12_H_12_]^2−^ show no interaction of this boron cluster with BSA. The measurements of fluorescence quenching were performed at a low stoichiometry of BSA and studied compounds, which was a maximum 1 : 1 for metallacarboranes and their derivatives (**1** to **6**) and 1 : 10 for other boron cluster compounds (**7** to **11**). Further studies with [CoD]^−^ and [B_12_H_12_]^2−^, using CD, DLS and SLS measurements as well as the MALDI-MS technique, were performed at a high stoichiometry up to 1: 1000. The results of these studies showed the non-specific binding of selected boron clusters with the BSA surface. Interactions of BSA with [CoD]^−^ are much stronger and have a bigger influence on the conformation of the protein than with [B_12_H_12_]^2−^. [CoD]^−^ specifically binds to the hydrophobic cavity of BSA at low stoichiometry and non-specifically binds with the surface of the protein at high stoichiometry. [B_12_H_12_]^2−^ interacts very weakly with BSA and only in the non-specific way at a high stoichiometry.

## Methods

### Materials and equipment

Cs[3-cobalt bis(1,2-dicarbollide)] and sodium dodecaborate, Na_2_[B_12_H_12_] (**11**), were purchased from Katchem (Prague, Czech Republic). Cs[3-iron bis(1,2-dicarbollide)] (**5**) was a kind gift from Prof. Jaromir Plešek (1927–2010). Fatty acid free BSA was purchased from Sigma-Aldrich (US), and dialysis tubes (Visking, MWCO 12–14 kDa) were from Serva GmbH, Germany. Sodium bicarbonate, analytical grade, was obtained from Avantor Performance Materials (Poland). All other chemicals were purchased from Sigma-Aldrich (US) and used without further purification. All solutions were prepared in Milli-Q water (18.2 M cm^−1^) produced by a Direct-Q3 UV system (Millipore, US). Absorption, fluorescence and circular dichroism (CD) spectra were recorded at 25 °C on J-1500 spectropolarimeter (Jasco, Japan) equipped with thermostated cell holder, FDT-538 fluorescence emission detector and PML-534 FDCD detector. For thermodynamic studies, fluorescence spectra were also recorded at 31 and 37 °C. Measurements were performed in rectangular quartz cuvettes with 5 mm and 1 mm optical path lengths for fluorescence and circular dichroism, respectively. UV-Vis spectra were recorded on a Specord 250 (Analytic Jena, Germany) spectrophotometer at ambient temperature. UV-Vis spectra were recorded in the range 250–500 nm with a slit width of 1 nm and scan speed of 10 nm s^–1^ in quartz cuvettes with a 5-mm optical path length. Dynamic and static light scattering (DLS and SLS) measurements were conducted on Zetasizer Nano ZS (Malvern Instruments, UK) with a 633-nm laser in a 12-μL quartz cuvette at a constant scattering angle of 173° in 0.1 M NaHCO_3_. MALDI-TOF MS analysis were performed on the ultrafleXtreme (Bruker Daltonics, Germany) in the linear positive ion mode at the laser frequency of 200 Hz. Calibrations were performed on protein calibration standard II from Bruker company.

### Chemistry

Cesium salt of Cs[3-cobalt bis(1,2-dicarbollide)] was converted to sodium salt (**1**) according the published procedure^56^ using the cationic exchange resin (Amberlite IR120, Acros Organics, US). Compounds **2**–**4** and **6**–**10** were obtained according to literature methods, as follows: 3-O-{{5-[3-cobalt-bis(1,2-dicarbollide)-8-yl]-3-oxa-pentoxy}-propyne (**2**)^57, 58^; 2-O-{{5-[3-cobalt-bis(1,2-dicarbollide)-8-yl]-3-oxa-pentoxy}-N-methylamine (**3**) synthetized *via* ring opening reaction of 8-dioxane-3-cobalt bis(1,2-dicarbollide) with CH_3_NH_2_^17, 59^. 2′-O{5-[3-Cobalt bis(1,2-dicarbollide)-8-yl]-3-oxa-pentoxy}-1N-1,2,3-triazole-4-yl}(4-propan-1-yl) adenosine (**4**) and 2′-O-{{5-[3-iron bis(1,2-dicarbollide)-8-yl]-3-oxa-pentoxy}-1N-1,2,3-triazole-4-yl}methyladenosine (**6**) were obtained according to previously published methods^60^; detailed experimental procedures will be described elsewhere. 1-(3-Bromopropanyl)-1,12-dicarba-closo-dodecaborane (**7**) and 1-(3-aminopropanyl)-1,12-dicarba-closo-dodecaborane (**9**) were synthesized following the method described earlier for the analogous derivatives of 1,7-dicarba-*closo*-dodecaborane^61^. 1-Carboxyl-1,12-dicarba-closo-dodecaborane (**8**) was obtained as described^62^ as was 2′-O-{[3-propyl-(1,12-dicarba-closo-dodecaboran-1-yl)]-1-N-1,2,3-triazol-4-yl}propyladenosine (**10**)^63^.

### Sample preparation

The stock solutions of the compounds **1**–**11** (10 mg mL^−1^) were prepared by dissolving the exact amounts in DMSO (compounds **2**–**10**) or 0.1 M NaHCO_3_ solution (compounds **1** and **11**). The stock solution of BSA (0.67 mM) was prepared using dialysis with 0.1 M sodium bicarbonate (pH 8.4), and its concentration was determined by the measurement of the absorbance at λ = 278 nm using ε = 44300 M^−1^cm^−1 64^. The BSA samples with boron clusters for fluorescence, UV-Vis, CD and light scattering analysis were prepared as follows: the stock solution of selected boron clusters was added to the BSA solutions in the desired molar ratio and measurements were taken immediately after mixing.

## Experimental procedure

### UV-Vis measurements

In all measured samples, BSA was diluted with 0.10 M sodium bicarbonate (pH 8.4) to a final concentration 10.5 × 10^−6^ M. Boron clusters **1** to **6** were added to the samples so the final concentration was 0 to 10.5 × 10^−6^ M. Boron clusters **7** to **11** were added to the sample so the final concentration was 0 to 10.5 × 10^−5^ M. In all samples, the concentration of DMSO was 2%. Absorption spectra were recorded from 250 to 500 nm. Each spectrum was corrected by the corresponding buffer blank.

### Fluorescence measurements

For fluorescence measurements, the samples were prepared in the same way as for UV-Vis measurements. Fluorescence emission spectra were recorded from 300 to 500 nm at three different temperatures (25, 31 and 37 °C with 3 min equilibrium time at each measurement temperature). The excitation wavelength was set at 280 nm. The data integration time was set to 1 s and HT to 600 V. An appropriate buffer blank spectrum was subtracted from the measured spectra for fluorescence background correction. The fluorescence intensity was corrected using the following equation:

7 $${F}_{corr}={F}_{obs}\times {e}^{\frac{{A}_{ex}+{A}_{ex}}{2}}$$

where *F*_*corr*_ and *F*_*obs*_ are the corrected and observed fluorescence intensities, respectively and *A*_*ex*_ and *A*_*em*_ are the absorbances at the excitation and emission wavelengths, respectively. The emission wavelength was 355 nm at the maximum of the fluorescence intensity spectrum. All fluorescence intensities used for the calculations were corrected in this work.

### Circular dichroism measurements

The BSA concentration was 1.5 μM for far UV measurements and 150 μM for near UV, and the final concentration of NaHCO_3_ was 10 mM for all CD measurements. Six spectra (recorded with a data pitch of 0.2 nm, band width of 1 nm, and data integration time of 2 sec at 50 nm min^−1^) were averaged for each sample. Each measurement was subtracted by NaHCO_3_ (10 mM) supplemented with a suitable amount of selected boron cluster. Therefore, the emerging spectrum is due to the BSA contribution alone. The CD signals were converted to the mean residue molar ellipticities using the mean residue weight of 114.2. The fractional contents of α-helices were calculated from far-UV CD spectra by the Dichroweb platform (CDSSTR with dataset 6).

### Dynamic and static light scattering measurements (DLS and SLS)

The final protein concentration was in the range 15.1–197 μM. The following parameters were used: protein refractive index (1.450) and solvent viscosity (NaHCO_3_, 0.1 M, 0.909 10^−4^ Pa×s). From three to six consecutive measurements of each sample were performed with an acquisition time of 30 s per correlation function. Scattering intensities for SLS analysis were derived from the average count rate of the samples and were calibrated against toluene using the Rayleigh ratio of R = 1.35 × 10^−5^cm^−1^. The refractive index increment with the BSA concentration at λ = 633 nm dn/dc_BSA_ = 0.185 was used. Data were analysed using dts 6.10 software (Malvern Instruments, orcestershire, UK). Particle-size distributions were obtained using the General Purpose algorithm included in the DTS software. In addition, the effect of temperature on the hydrodynamic parameters of BSA in the presence of boron clusters was estimated by DLS at 5 °C intervals from 25 to 85 °C and a 3-min equilibrium time at each measurement temperature.

### MALDI-TOF MS

The matrix, 2,5-dihydroxybenzoic acid (DHB, Sigma), was dissolved in CH_3_CN : H_2_O : CF_3_COOH (50 : 50 : 0.1) to a final concentration of 50 mg mL^−1^. Protein samples containing 15 μM BSA and selected boron clusters in 0.1 M NaHCO_3_ solution were diluted with equal volumes of matrix solution and were evaporated (0.5 μL) on a steel plate at room temperature.

### Data Availability

All data generated or analysed during this study are included in this published article (and its Supplementary Information files).

## Electronic supplementary material

Supplementary Figures and Tables
