# Supplementary material for: Interactions of Boron Clusters and their Derivatives with Serum Albumin
Source: Sci Rep. 2017 Aug 29;7:9800. doi: 10.1038/s41598-017-10314-0 (PMC5574927; doi:10.1038/s41598-017-10314-0)
Supplement: Supplementary file 1 — Supplementary Figures and Tables [file 41598_2017_10314_MOESM1_ESM.pdf]

Supplementary Figures and Tables

Interactions of Boron Clusters and their Derivatives with Serum Albumin

Tomasz M. Goszczyński[a]*, Krzysztof Fink[a], Konrad Kowalski[a], Zbigniew J. Leśnikowski[b]* and Janusz Boratyński[a]

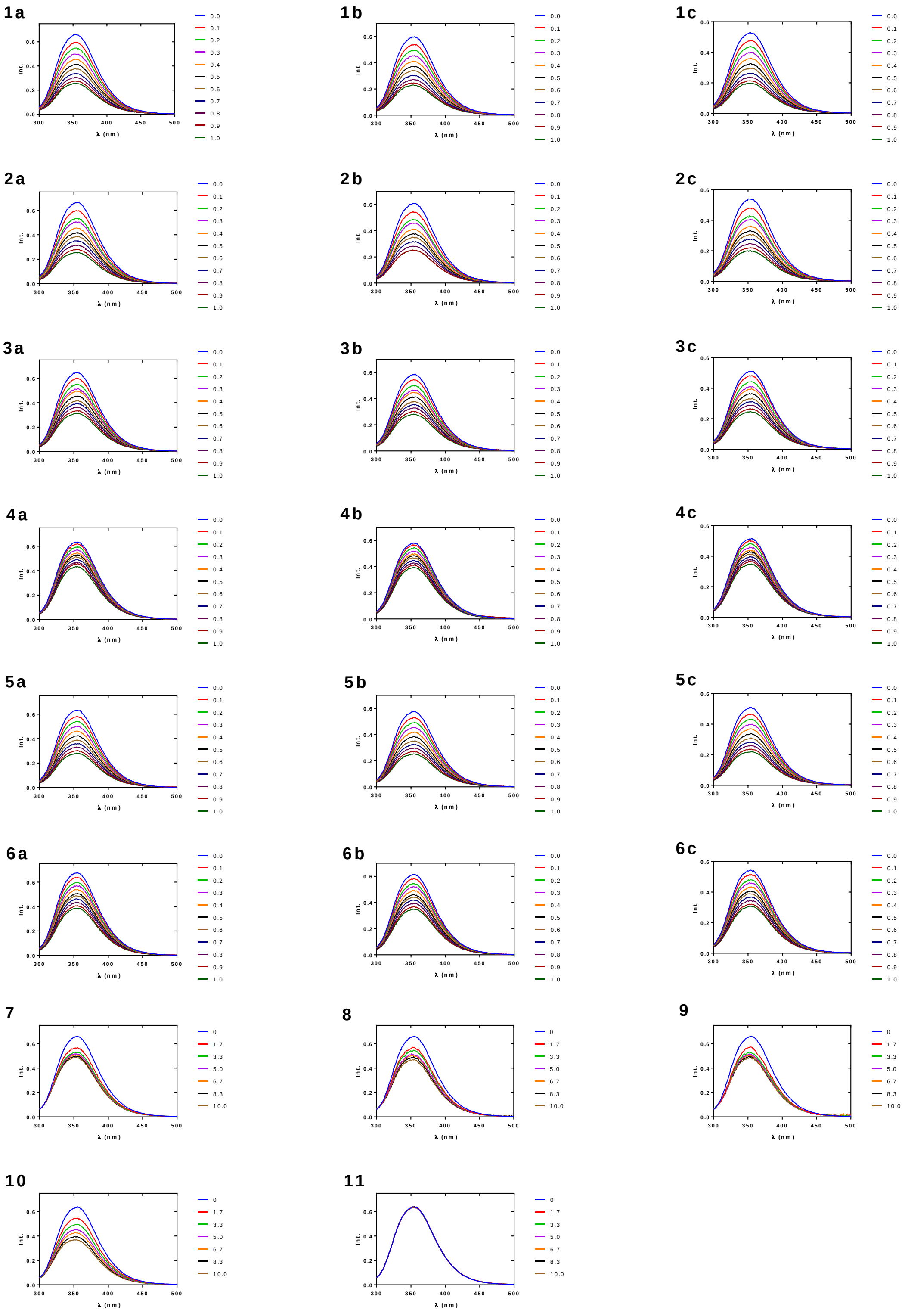

Figure S1. Fluorescence emission spectra of BSA (10.5 µM) in the presence of boron cluster and their derivatives in 0.10 M sodium bicarbonate (pH 8.4) with 2% DMSO, λ_{ex}=280 nm. Numbers **1** to **11** are symbols of the boron compounds, whereas letters **a**, **b** and **c** show the temperature at which the measurement was performed (25, 31 and 37°C, respectively). Measurements for compounds **7** to **11** were performed only at 25°C. The concentration of **1** to **6** was 0 to 10.5 µM with 1.05 µM intervals; the concentration of **7** to **11** was 0 to 105 µM with 17.5 µM intervals. Detailed molar excess of boron cluster is described in the legend.

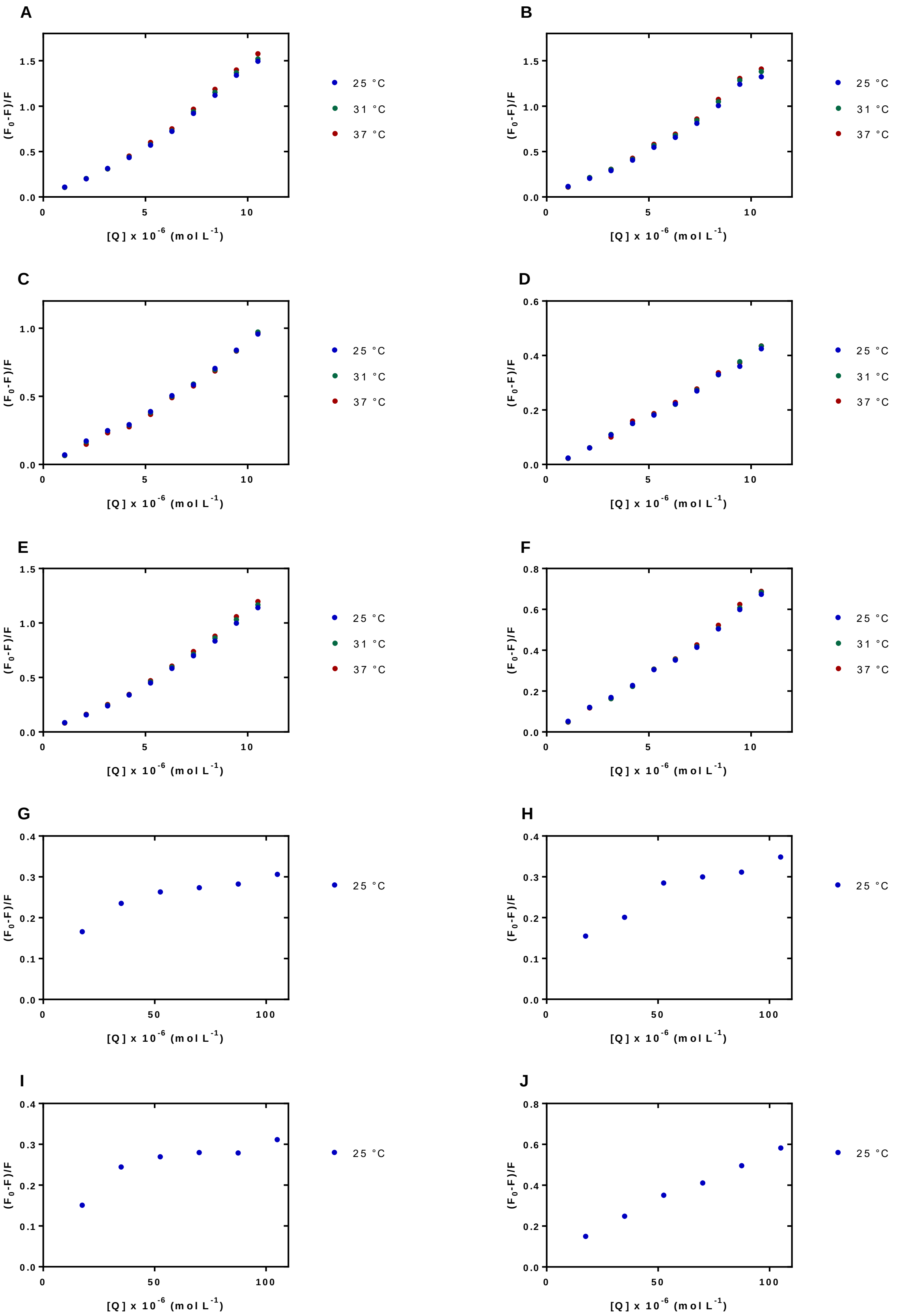

Figure S2. Stern-Volmer plots for the binding of BSA with compounds **1** (A), **2** (B), **3** (C), **4** (D), **5** (E), **6** (F), **7** (G), **8** (H), **9** (I) and **10** (J) at various temperatures (25, 31 and 37°C).

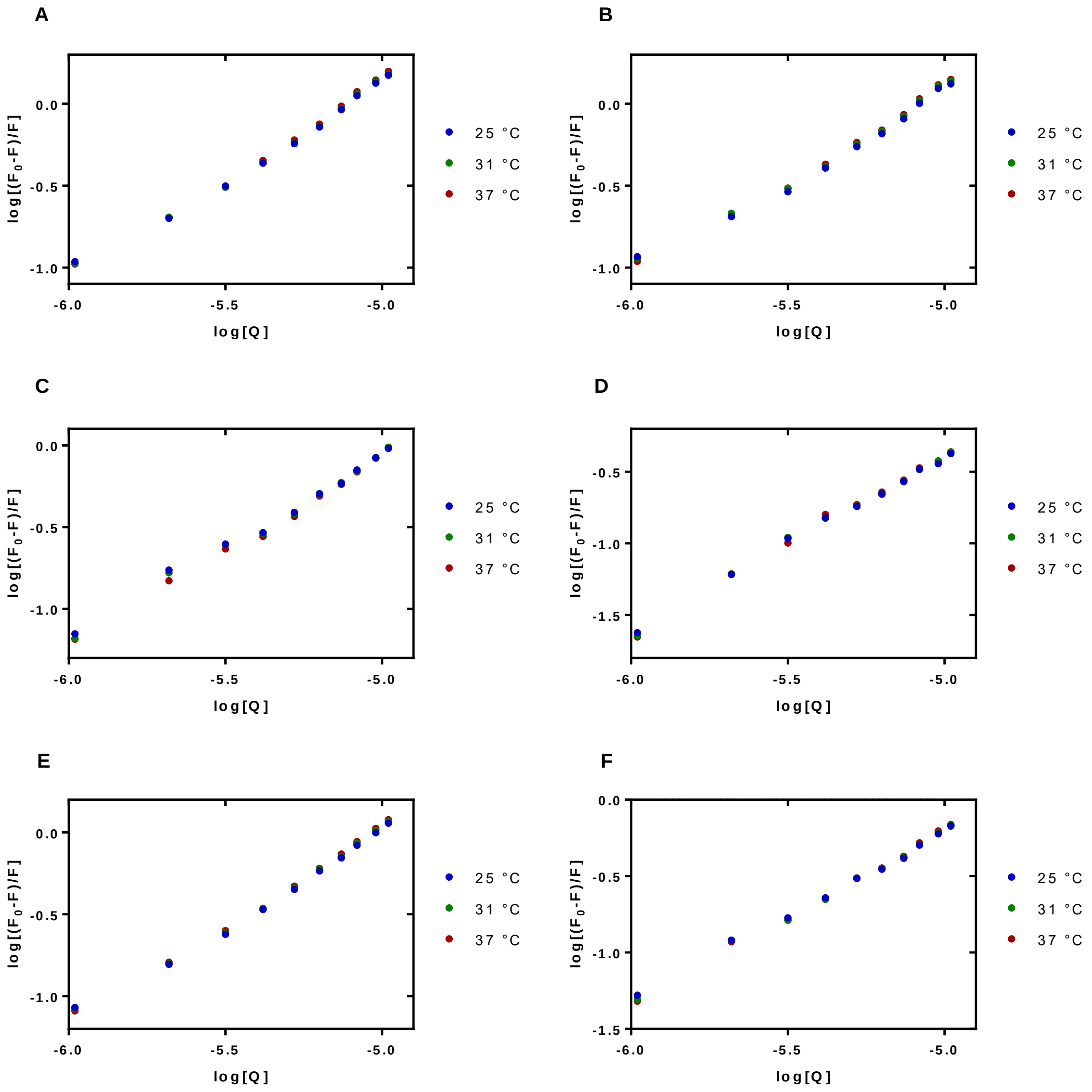

Figure S3. Double-logarithmic plots for the binding of BSA with compounds **1** (A), **2** (B), **3** (C), **4** (D), **5** (E) and **6** (F) at various temperatures (25, 31 and 37°C).

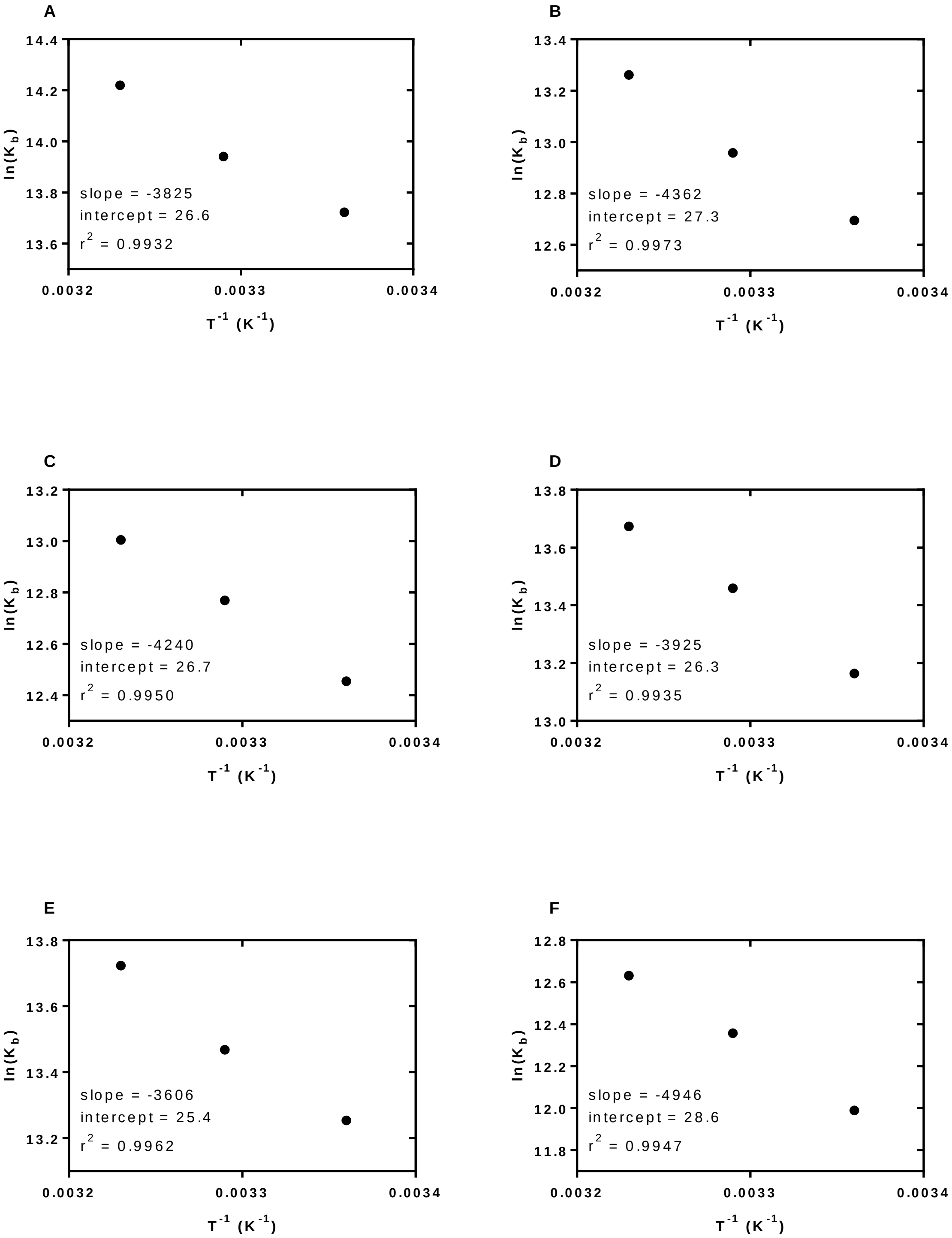

Figure S4. Van't Hoff plots for the binding of BSA with compounds **1** (A), **2** (B), **3** (C), **4** (D), **5** (E) and **6** (F).

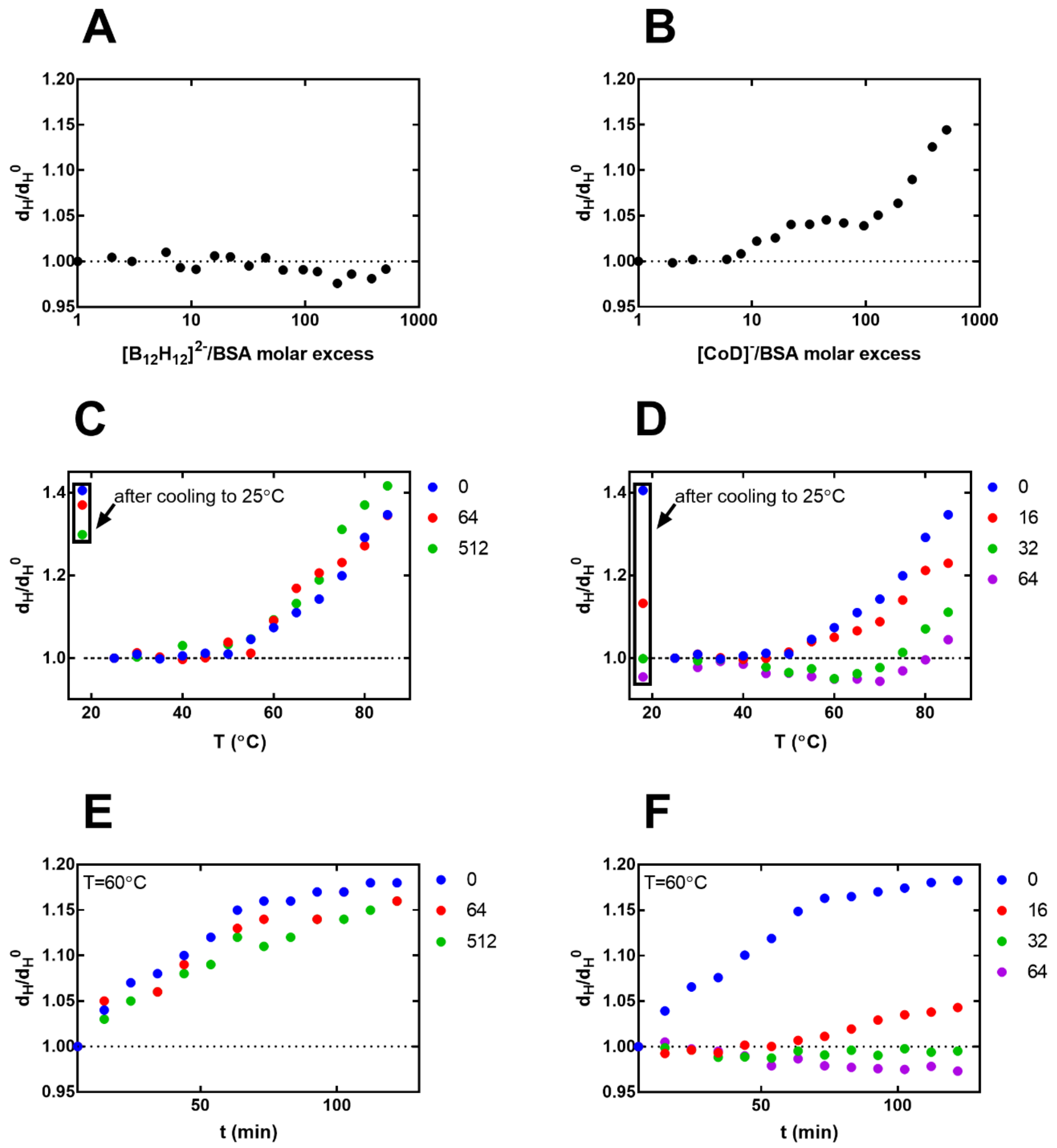

Figure S5. Relative hydrodynamic diameter of BSA determined by the DLS method: as a function of concentration of $[B_{12}H_{12}]^{2-}$ (A) and $[CoD]^-$ (B), where $d_H{}^0$ is the hydrodynamic diameter of BSA without boron clusters addition, boron clusters concentration were in the range 0-7.7mM; as a function of temperature with specified in the legend $[B_{12}H_{12}]^{2-}$/BSA (C) and $[CoD]^-$/BSA (D) molar ratio where $d_H{}^0$ is the hydrodynamic diameter of BSA with or without boron cluster in 25°C; as a function of time (kinetics of thermal denaturation of BSA)) at 60°C with specified in the legend $[B_{12}H_{12}]^{2-}$/BSA (E) and $[CoD]^-$/BSA (F) molar ratio, where $d_H{}^0$ is the initial value of hydrodynamic diameter of BSA with or without boron cluster (after 5min incubation at 60°C). All measurement were made in $NaHCO_3$ (0.1M) with constant concentration of BSA (15μM).

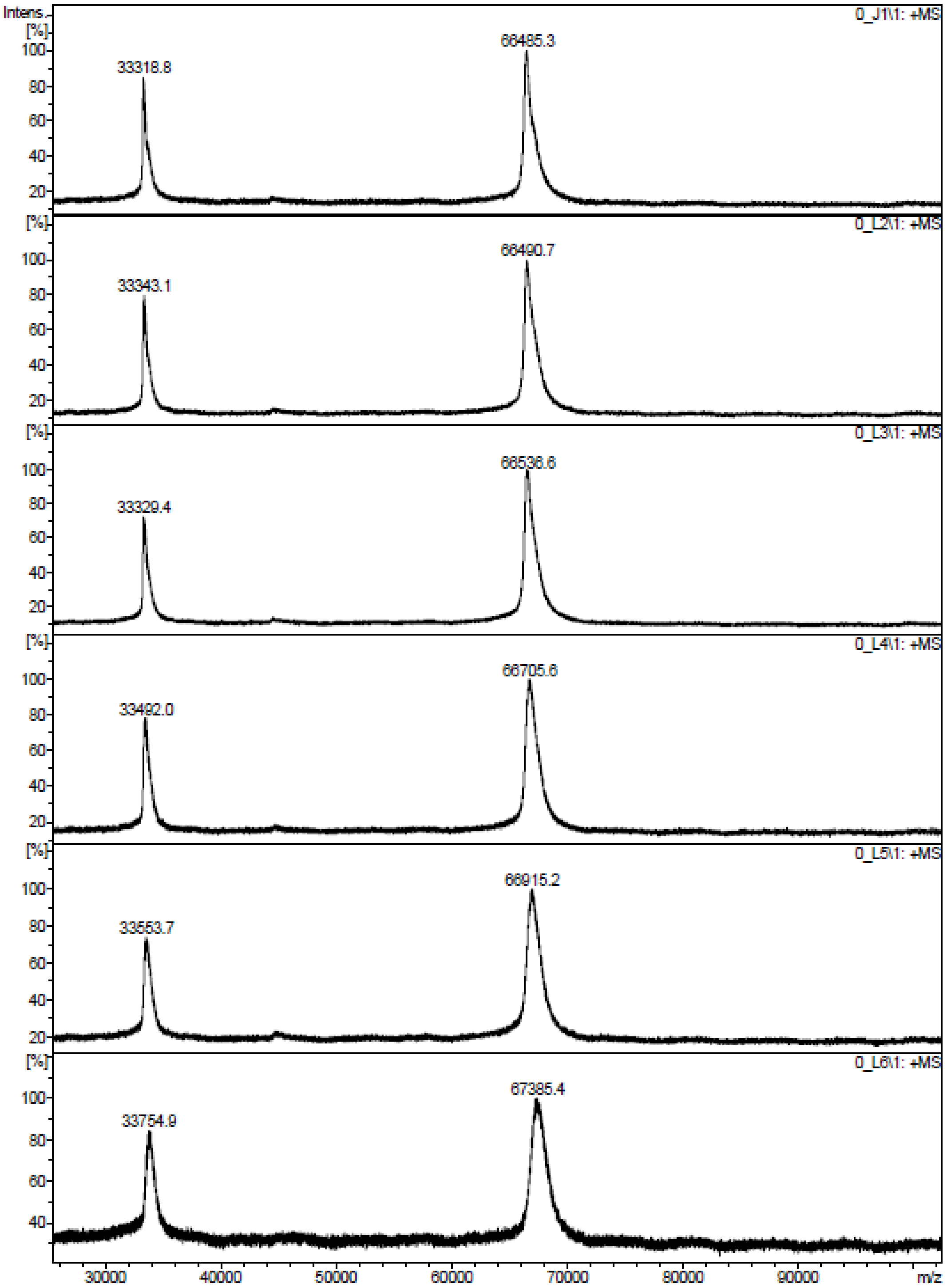

Figure S6. Linear positive MALDI mass spectra of nonconvalent binding complexes of BSA and $[B_{12}H_{12}]^{2-}$.

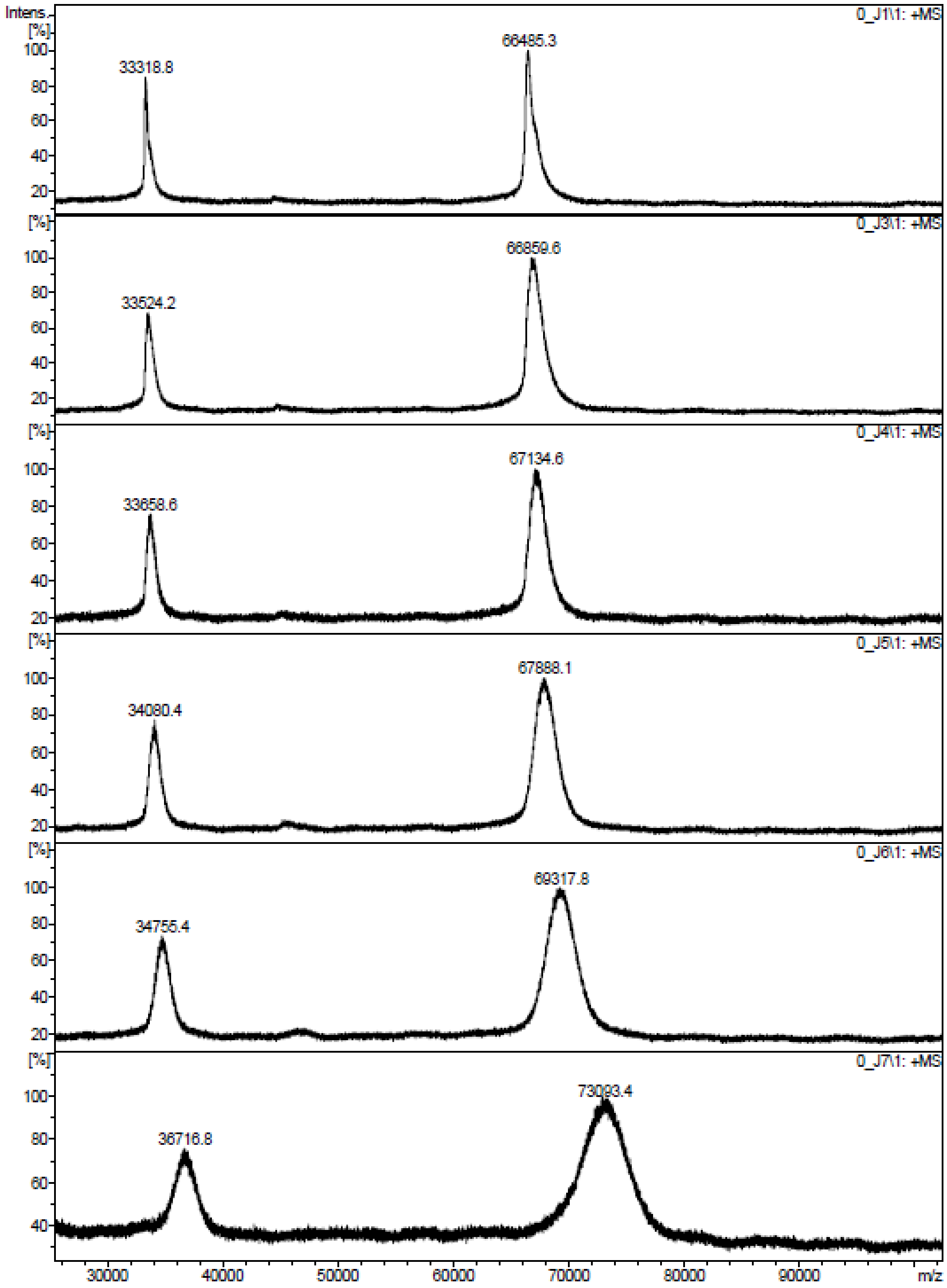

Figure S7. Linear positive MALDI mass spectra of nonconvalent binding complexes of BSA and $[CoD]^-$.

Table S1. Degree of quenching of: **1** – metallacarborane, **7** – *para*-carborane and **11** – dodecaborate. Fluorescence emission spectra for BSA (10.5 μM) in the presence of boron clusters in 0.10 M sodium bicarbonate (pH 8.4) with 2% DMSO was recorded from 300 to 500 nm, λ_{ex}=280 nm, at 298 K. The concentration of boron clusters was 10.5 μM. Equation for calculation of degree of quenching parameter, where F and F_0 are the steady-state fluorescence intensities in the presence and absence of quencher.

Compound	Degree of quenching [%]	Equation
**1**	60	
**7**	6	$degree\ of\ quenching = (1 - \frac{F}{F_0}) \cdot 100\%$
**11**	0	

Table S2. Stern-Volmer constant (K_{SV}) and quenching constant (k_q) for interaction of boron clusters (**1** to **11**) with BSA at 298, 304 and 310 K. Fluorescence emission spectra of BSA (10.5 μM) in the presence of boron clusters (**1** to **11**) in 0.10 M sodium bicarbonate(pH 8.4) with 2% DMSO, at λ_{ex}=280 nm was recorded. The concentration of **1** to **6** was 0 to 10.5 μM with 1.05 μM intervals; the concentration of **7** to **11** was 0 to 105 μM with 17.5 μM intervals.

Compund	T (K)	K_{SV} (M^{-1})	k_q (M^{-1} s^{-1})	r^2
	298	(1.51 ± 0.07) × 10^5	2.51 × 10^{13}	0.9838
**1**	304	(1.54 ± 0.07) × 10^5	2.57 × 10^{13}	0.9831
	310	(1.60 ± 0.07) × 10^5	2.67 × 10^{13}	0.9841
	298	(1.33 ± 0.07) × 10^5	2.22 × 10^{13}	0.9808
**2**	304	(1.39 ± 0.07) × 10^5	2.31 × 10^{13}	0.9812
	310	(1.42 ± 0.07) × 10^5	2.37 × 10^{13}	0.9828
	298	(9.19 ± 0.37) × 10^4	1.53 × 10^{13}	0.9874
**3**	304	(9.30 ± 0.40) × 10^4	1.55 × 10^{13}	0.9856
	310	(9.34 ± 0.43) × 10^4	1.56 × 10^{13}	0.9836
	298	(4.16 ± 0.10) × 10^4	6.94 × 10^{12}	0.9953
**4**	304	(4.28 ± 0.12) × 10^4	7.14 × 10^{12}	0.9942
	310	(4.32 ± 0.10) × 10^4	7.19 × 10^{12}	0.9959
	298	(1.13 ± 0.04) × 10^5	1.88 × 10^{13}	0.9880
**5**	304	(1.17 ± 0.05) × 10^5	1.94 × 10^{13}	0.9875
	310	(1.20 ± 0.05) × 10^5	2.00 × 10^{13}	0.9878
	298	(6.48 ± 0.21) × 10^4	1.08 × 10^{13}	0.9915
**6**	304	(6.62 ± 0.23) × 10^4	1.10 × 10^{13}	0.9906
	310	(6.79 ± 0.23) × 10^4	1.13 × 10^{13}	0.9911
	298			
**7 - 11**	304	n.d.		
	310			

Table S3. Binding parameters (binding constant – K_b and binding sites – n) and thermodynamic parameters (Gibbs free energy - ΔG^0, enthalpy - ΔH^0 and entropy - ΔS^0) for interaction of boron clusters (**1** to **11**) with BSA at 298, 304 and 310 K. Fluorescence emission spectra of BSA (10.5 µM) in the presence of boron clusters (**1** to **11**) in 0.10 M sodium bicarbonate (pH 8.4) with 2% DMSO, at λ_{ex}=280 nm was recorded. The concentration of **1** to **6** was 0 to 10.5 µM with 1.05 µM intervals; the concentration of **7** to **11** was 0 to 105 µM with 17.5 µM intervals.

	T (K)	logK_b	n	r^2	K_b (M^{-1})	$\Delta G^0{}_1$[a] (kJ mol^{-1})	$\Delta G^0{}_2$[b] (kJ mol^{-1})	ΔH^0 (kJ mol^{-1})	ΔS^0 (J mol^{-1} K^{-1})
	298	5.96 ± 0.21	1.17 ± 0.04	0.9906	9.11×10^5	-34.0	-34.0	31.8	221
**1**	304	6.05 ± 0.21	1.19 ± 0.04	0.9910	1.13×10^6	-35.3	-35.2		
	310	6.18 ± 0.21	1.21 ± 0.04	0.9918	1.50×10^6	-36.6	-36.6		
	298	5.51 ± 0.25	1.09 ± 0.05	0.9853	3.26×10^5	-31.4	-31.4	36.3	227
**2**	304	5.63 ± 0.23	1.11 ± 0.04	0.9885	4.24×10^5	-32.8	-32.8		
	310	5.76 ± 0.20	1.13 ± 0.04	0.9910	5.75×10^5	-34.2	-34.2		
	298	5.41 ± 0.18	1.10 ± 0.03	0.9927	2.56×10^5	-30.9	-30.9	35.2	222
**3**	304	5.55 ± 0.18	1.12 ± 0.03	0.9929	3.51×10^5	-32.2	-32.3		
	310	5.65 ± 0.17	1.14 ± 0.03	0.9941	4.44×10^5	-33.5	-33.5		
	298	5.72 ± 0.14	1.22 ± 0.03	0.9966	5.21×10^5	-32.6	-32.6	32.6	219
**4**	304	5.85 ± 0.16	1.25 ± 0.03	0.9955	7.00×10^5	-34.0	-34.0		
	310	5.94 ± 0.15	1.26 ± 0.03	0.9959	8.67×10^5	-35.3	-35.2		
	298	5.76 ± 0.20	1.15 ± 0.04	0.9919	5.70×10^5	-32.8	-32.8	30.0	211
**5**	304	5.85 ± 0.19	1.17 ± 0.04	0.9926	7.06×10^5	-34.1	-34.0		
	310	5.96 ± 0.17	1.19 ± 0.03	0.9945	9.11×10^5	-35.4	-35.4		
	298	5.21 ± 0.11	1.08 ± 0.02	0.9970	1.61×10^5	-29.7	-29.7	41.1	238
**6**	304	5.37 ± 0.13	1.12 ± 0.02	0.9960	2.33×10^5	-31.2	-31.2		
	310	5.49 ± 0.12	1.14 ± 0.02	0.9967	3.06×10^5	-32.6	-32.6		
	298								
**7-11**	304	n.d.							
	310								

[a] $\Delta G_1 = \Delta H - T\Delta S$

[b] $\Delta G_2 = \mathrm{RTln}(K_b)$
